# Editorial for ‘New talent’ collection: introducing New Talent in chemistry and material science

**DOI:** 10.1098/rsos.181141

**Published:** 2018-08-15

**Authors:** Jeremy K. M. Sanders, Anthony Stace

**Affiliations:** 1 Department of Chemistry, University of Cambridge, Cambridge, CB2 1EW, UK; 2 School of Chemistry, University of Nottingham, Nottingham, UK

**Keywords:** chemistry, collaboration, Royal Society Open Science, Royal Society of Chemistry, material science

## Abstract

We introduce 14 articles published as part of the ‘New talent’ special collection of invited articles to showcase some of the exciting work being funded by the Royal Society. As Royal Society University Research Fellows or Dorothy Hodgkin Fellowship holders, the contributors to this collection are rising stars in their areas of research. This collection also illustrates the close collaboration between *Royal Society Open Science* and the Royal Society of Chemistry. The collection spans the range of the chemical and material sciences, reflecting the breadth of research areas receiving Royal Society grant support.

## 1. Introduction

*Royal Society Open Science* is unique in the portfolio of journals published by the Royal Society. It was the first to operate open peer review at the Society and is our first truly cross-disciplinary journal. It is also the first to be published in collaboration with another learned academic Society, the Royal Society of Chemistry (RSC). This collection of articles has its genesis in that collaboration and in the support the Royal Society provides to new talent through its University Research and Dorothy Hodgkin Fellowships.

To the best of our knowledge, the collaboration is unique in scientific scholarly publishing. Under many circumstances, two academic Societies, each with interests in publishing chemistry research content, would not be collaborators but competitors. The goal of the collaboration is to provide a joint outlet for the two Societies to allow them to publish high-quality chemistry and material science research, regardless of its subjective impact.

Since 2015, the peer review of all chemistry content submitted to *Royal Society Open Science* has been managed by the RSC.^1^ A subset of the journal's Associate Editors with expertise in chemistry, led by Anthony Stace as Subject Editor, work with the RSC to conduct the peer review of papers submitted to *Royal Society Open Science* in chemistry and allied fields such as material science. Any paper eventually accepted is published on the journal's website, and includes an RSC badge to acknowledge the collaboration.

The publications in this collection each include a holder of a Royal Society University Research Fellowship or a Dorothy Hodgkin Fellowship. These awards are granted via a competitive grant application process by the Royal Society. University Research Fellowships are awarded to ‘outstanding scientists who are in the early stages of their research career and have the potential to become leaders in their field’.^2^ Dorothy Hodgkin Fellowships, meanwhile, are awarded to equally promising early career researchers who require additional flexibility in their working patterns, owing to their personal circumstances.^3^

Inviting these future leaders to submit to *Royal Society Open Science* was a strong endorsement of the high quality of the research these individuals are conducting. Furthermore, the curation of this ‘New talent’ special collection provided an opportunity for the journal to work closely with the wider Royal Society to establish a ‘proof of principle’ for future ‘New talent’ collections in other major areas of research. Lastly, and to reiterate, the collection provides a vindication of the opportunities presented when two learned Societies work on a joint venture providing a template for new collaborations in future.

The collection comprises 14 papers, curated at http://royalsocietypublishing.org/cc/new-talent.^4^ The contributions span across the range of theoretical and experimental chemical and material sciences, both original research and reviews, reflecting the breadth of research supported by the Royal Society. As readers will see, the collection represents a wide swathe of chemical research from inorganic to metalorganic to biological and analytical chemistry.

We have enjoyed reading and preparing these manuscripts for publication, and we hope that readers of the journal will also find something of interest in these papers. As Editors of the journal, we are grateful for the support provided by these Fellows, and we hope this is the beginning—rather than the conclusion—of continuing relationships between them and *Royal Society Open Science*.

## Footnotes

1 *Royal Society Open Science*. Chemistry (accessed 4 June 2018). http://rsos.royalsocietypublishing.org/chemistry/.

2 The Royal Society. University Research Fellowship (accessed 4 June 2018). https://royalsociety.org/grants-schemes-awards/grants/university-research/.

3 The Royal Society. Dorothy Hodgkin Fellowship (accessed 4 June 2018). https://royalsociety.org/grants-schemes-awards/grants/dorothy-hodgkin-fellowship/.

4 *Royal Society Open Science*. Royal Society Open Science ‘New talent’ (accessed 12 July 2018). http://royalsocietypublishing.org/cc/new-talent.

## Data accessibility

This article has no additional data.

